# Obstetrics and Gynecology

**Published:** 1893-08

**Authors:** 


### OBSTETRICS AND GYNECOLOGY.

EDITED BY DR. VIRGIL O. HARDON.

### ENGSTRÖM: THE ETIOLOGY OF TUBAL PREGNANCY (*Zeitschrift für Geburtshülfe und Gynäkologie*).

In two cases of tubal pregnancy which he had examined before impregnation occurred, E. demonstrated a diseased condition (thickening) of the affected tubes, a mild degree of pelveo-peritonitis, fixation of the ovaries through pseudo-membranes, and a uterine catarrh. It is certain that the ciliated epithelium of the Fallopian tubes was more or less damaged, and consequently its power to transport the ovum into the uterine cavity was diminished. The cause of ectopic gestation must be sought for in an obstructed passage of the ovum from the ovaries through the tubes into the uterus. The ovum, after its discharge from the Graafian follicle, is swept by the ciliary epithelium into the Fallopian tubes and uterus. Peristaltic contraction of the tube may aid this passage. Modern investigations no longer justify the belief that the ovum can pass from one tube through the cavity of the uterus into another tube.

### INTRAUTERINE THERAPEUTICS.

Mackenrodt (*Samml. Klin. Vorträge, n. f.* No. 45) relates his experience for a period of two years with curettage and intrauterine injections. The results of forty-five cases occurring during the last half year are given somewhat in detail. Of these, seventeen cases were cured, twenty-two were improved, five were made no better and withdrew from treatment. The patients were treated for the most part in the ambulance service, and the injections were made at first every other day, but later on only twice weekly. According to the author, all affections of the uterus or of the adnexa which commence primarily as an endometritis should be treated with intrauterine medication. But the acute stage must be over. Affections of the adnexa and their surroundings are not contraindications of intrauterine treatment, as is held by some; on the contrary they are indications for the treatment, only one should be careful in carrying it out not to dislocate the uterus. Intrauterine treatment is indicated in the following affections: Endometritis, metritis, and perimetritis chronica, parametritis and parametritis posterior chronica, oöphoritis and perioöphoritis subacute and chronic. In cases of pyosalpinx and purulent ovarian tumors the treatment is of no avail.—*N. Y. Journal of Gynecology.*

---

### PELZER: A METHOD TO PRODUCE PREMATURE LABOR BY INTRA-UTERINE INJECTIONS OF GLYCERIN (*Archiv für Gynäkologie*).

The method has been in practice in the Cologne Maternity Hospital since July, 1890, both in cases of premature labor and when the labor pains are feeble and ineffective. The author claims that it is a decided improvement over the older methods. The mode of operation is as follows: The patient is placed in the knee-elbow or Sims' position to prevent the escape of the injected glycerin. A balloon or piston syringe with a long nozzle, curved to correspond to the contour of the uterus, is introduced between the membranes and the uterine walls, and about one hundred and fifty grammes of pure glycerin are slowly injected. Care must be observed that the syringe is freed from air bubbles, which might be forced into the uterine sinuses.

The glycerin produces uterine contractions:

1. By means of the direct irritation of the inner surface of the uterus.

2. Through a mechanical separation of the membranes from the uterine walls.

3. Owing to its hygroscopic action a transudation of the liquor amnii is caused, and the ovum, becoming smaller, is consequently detached from the uterine walls.

Besides being prompt in its action, glycerin is an antiseptic medium and also lubricates the genital canal.

### A Few General Principles Relating to the Treatment of Endometritis and to Complications of the Ovary and Tubes (*Gaz. de. Gyn.*)

In conclusion Boulangier sums up as follows:

1. The therapeusis of affections of the uterine appendages is the same as that of uterine affections.

2. In acute cases of pelvic peritonitis or ovarosalpingitis we should begin with conservative measures, consisting in energetic antiseptic and antiphlogistic treatment.

3. The one exception to this rule consists in acute peritonitis from presumed rupture of a purulent mass. Laparotomy should be resorted to without delay.

4. In subacute cases with relapses and intermittent febrile movement, conservative therapeusis (curettage, dilatation, disinfection, tampons, hot douches, salicylates, an iodides) is usually efficacious.

5. If the disease persists and abscesses become localized, recourse should be had to surgical intervention.

Major operations should be reserved for chronic cases. But a complete sacrifice of the organ should not be made until it is certain that less destructive methods are of no avail. As the president of the recent Parisian Surgical Congress remarked: "When we have the choice of two operations we should not choose the more serious one in order to gratify our pleasure in overcoming difficulties, or in order to place a false aureole upon our brows."

---

### Effects of Morphine on the Female Organs.

Passower (*Centralbl. f. Gynæk*, No. 2, 1893) recently read a paper before the Obstetric Society of St. Petersburg, in which he related the course of two cases under his own observation. It confirmed an opinion already supported by the observation of others, that the abuse of morphine eventually leads to atrophy of the female organs. Passower's cases were of the ages of twenty-nine and thirty. One consulted him on account of the resultant amenorrhea. The drug was discontinued, and the catamenia reappeared. The patient took to morphine again, and straightway the menses ceased. Between

1887 and 1889 Passower observed the case. Sixteen pounds weight was lost, and the subcutaneous fat disappeared. The vulva atrophied. The measurements of the uterus during that period ran as follows: December, 1887, three and one-tenth inches; May, 1888, two and nine-tenths inches; November, 1888, two and seven-tenths inches; April, 1889, two and three-fifths inches; September, 1889, two and three-tenths inches; and July, 1890, one and nine-tenths inches. The atrophic process no doubt began in the ovaries and spread to the other parts of the genital tract. This is evident from the early appearance of amenorrhea and the later atrophy of the vulva, and also from physiological evidence. Thus the submaxillary glands atrophy in dogs subjected to doses of morphine. How much of the drug can be taken without danger of these ill effects is entirely an individual question.—*British Medical Journal.*

---

### THE BEST DRESSING FOR THE UMBILICAL CORD.

It is very generally admitted that the detachment of the stump of the umbilical cord in infants is retarded by the application of an antiseptic dressing. The cord drops off on the fifth or sixth day if it is simply wrapped up in a piece of rag greased with oil or lard —as used to be practiced before the introduction of the antiseptic system—whereas it may not become detached until the tenth day when a dry antiseptic dressing is used, such, for instance, as dusting with a mixture of the subnitrate of bismuth and iodoform.

Does this imply that the principles of the antiseptic method should be disregarded so far as the umbilical cord is concerned? Far from it, for although the old practice of dressing the cord with oil or lard answers the purpose very well in the vast majority of cases, yet it presents this disadvantage, that in some cases under its influence the stump is apt on dropping off to leave behind it a dirty unhealthy looking sore, which may become the starting point of various infective processes.

The ideal dressing would therefore be one which would not retard the detachment of the cord, while at the same time being sufficiently antiseptic to protect the infant from all risk of infection.

According to Dr. J. Lvow, Privat-docent of Obstetrics at the Medical Faculty of Kasan, this twofold object can be secured by the use of glycerine, which, besides being antiseptic, has also a distinct effect in promoting cicatrization. From the results obtained with this method in the case of more than five hundred infants, Dr. Lvow is satisfied that under the influence of glycerine the cord shrivels up and drops off on the fourth, or at latest, on the fifth day.

The following is the method employed by Dr. Lvow:

The cord having been tied and divided, and the child washed, the stump of the cord is carefully dried with a piece of cotton-wool, after which it is entirely covered with cotton-wool moistened with pure glycerine. The dressing is kept in position by means of a few turns of gauze bandage. It is left undisturbed until the cord becomes detached, that is to say, until the fourth or fifth day. During that time the child is not to be washed, and it is better to abstain even from examining the cord. One should be content with changing the gauze bandage when it is soiled.

By this means the cord on dropping off leaves behind it a healthy looking sore, perfectly free from exudation and inflammatory redness. The treatment is completed by the application of the subnitrate of bismuth, or of powdered talc, and healing is complete by the tenth day.—*Medical Week.*

---

### THE EFFECT OF COMPRESSION OF ABDOMINAL AORTA FOR THE RELIEF OF UTERINE HEMORRHAGE.

Bishop, in a lecture delivered at the Ancoats Hospital, Manchester (*Lancet,* 1893, No. xvi.), recommends compression of the aorta in cases of uterine hemorrhage as the quickest and most convenient manner of checking it. Time in this condition is of importance, because the longer hemorrhage lasts, the longer it is likely to continue on account of the general relaxation of the uterine tissue. The uterine arteries, through the internal and common iliac, start from a common source, the aorta, and can be controlled with the greatest ease by pressure upon it. The general condition of relaxation, and particularly of the abdominal muscles, from the bleeding, greatly favors this method of relief. The hand sinks into the abdomen without opposition until the sacral promontory is reached, and immediately above and to the left the main artery is discovered by its pulsations. He gives an interesting account of the pathology of uterine hemorrhage, and cites two cases of post-partum hemorrhage in which compression of the aorta was successfully used.—*Journal of American Medical Sciences.*

